# Investigation of Microstructures and Tensile Properties of 316L Stainless Steel Fabricated via Laser Powder Bed Fusion

**DOI:** 10.3390/ma17040913

**Published:** 2024-02-16

**Authors:** Melody Chepkoech, Gbadebo Owolabi, Grant Warner

**Affiliations:** 1 Department of Mechanical Engineering, Howard University, Washington, DC 20059, USA; gbadebo.owolabi@howard.edu; 2 Center for Black Entrepreneurship, Spelman College, Atlanta, GA 30314, USA

**Keywords:** 316L stainless steel, laser powder bed fusion, additive manufacturing, tensile, microstructure

## Abstract

In this study, a thorough investigation of the microstructures and tensile properties of 316L stainless steel fabricated via laser powder bed fusion (L-PBF) was done. 316L stainless steel specimens with two different thicknesses of 1.5 mm and 4.0 mm fabricated under similar conditions were utilized. Microstructural characterization was performed using optical microscopy (OM) and scanning electron microscopy (SEM) equipped with electron backscatter diffraction (EBSD). Melt pools and cellular structures were observed using OM, whereas EBSD was utilized to obtain the grain size, grain boundary characteristics, and crystallographic texture. The 1.5 mm thick sample demonstrated a yield strength (YS) of 538.42 MPa, ultimate tensile strength (UTS) of 606.47 MPa, and elongation to failure of 69.88%, whereas the 4.0 mm thick sample had a YS of 551.21 MPa, UTS of 619.58 MPa, and elongation to failure of 73.66%. These results demonstrated a slight decrease in mechanical properties with decreasing thickness, with a 2.4% reduction in YS, 2.1% reduction in UTS, and 5.8% reduction in elongation to failure. In addition to other microstructural features, the cellular structures were observed to be the major contributors to the high mechanical properties. Using the inverse pole figure (IPF) maps, both thicknesses depicted a crystallographic texture of {001} <101> in their as-built state. However, when subjected to tensile loads, texture transitions to {111} <001> and {111} <011> were observed for the 1.5 mm and 4.0 mm samples, respectively. Additionally, EBSD analysis revealed the pre-existence of high-density dislocation networks and a high fraction of low-angle grain boundaries. Interestingly, twinning was observed, suggesting that the plastic deformation occurred through dislocation gliding and deformation twinning.

## 1. Introduction

For several decades, conventional manufacturing methods, such as casting, have been the cornerstone of mass-producing cost-effective metal components. 316L stainless steel is one of the major materials mainly manufactured through casting. This type of stainless steel is commonly used in a variety of industrial applications, including medical, aerospace, and automotive applications [1]. It is known for its excellent corrosion resistance, high strength, and good weldability [2,3]. However, conventionally manufactured steel, particularly low-carbon steel produced through casting, is recognized for its characteristic of having relatively low yield strength [4,5].

According to Muley et al. [6], steels do not undergo a phase transformation; thus, heat treatment does not increase their strength. However, by refining their grain sizes, their mechanical properties can be enhanced. Traditionally, grain refinement is achieved through severe plastic deformation processes, such as cold rolling, equal channel angular pressing, or dynamic plastic deformation at high strain rates [7,8], or other methods, such as the introduction of bimodal grain sizes [9] and recrystallization [10]. Fabricating complex components with these methods can be quite challenging, as extensive machining and tooling processes are needed.

Laser powder bed fusion (L-PBF) has emerged as a promising method that is likely to revolutionize the manufacturing industry by enabling the fabrication of complex designs without necessarily requiring additional machining. Additionally, it enables the tailoring of the mechanical properties by allowing for the selection of fabrication parameters [11]. For traditionally manufactured 316L stainless steel, an increase in strength through grain refinement methods usually results in a decrease in ductility, hence the strength–ductility tradeoff [5]. However, recent studies showed that the use of the L-PBF method and the proper selection of fabrication parameters can result in the production of components with unique microstructural features that enhance both their strength and ductility [12,13].

Wang et al. [14] studied the mechanical properties of L-PBF 316L stainless steel. The authors observed an increase in both strength and ductility. The increase in strength and ductility was attributed to the combinatory contributions of cellular structures, low-angle grain boundaries, and dislocation networks. Suryawanshi et al. [15] attributed the increased yield strength of Selective Laser Melting (SLM) 316L stainless steel to the substantial refinement in the microstructures due to the rapid solidification process. According to Liu et al. [16], the increase in strength can be attributed to the segregation of alloying elements and the presence of dislocation network structures in SLM 316L stainless steel. These studies showed that additive manufacturing causes the formation of diverse microstructures that need to be studied further to gain a comprehensive understanding.

The continued prominence of the L-PBF process in the past few years can also be attributed to its ability to produce fully dense parts, with relative densities of up to 99.9% [11]. The high relative densities of L-PBF components make them suitable for a wide range of applications while at the same time increasing their adoption to the industry for various applications. Afkhami et al. [17] conducted a study on samples fabricated vertically and horizontally and identified that they were fully dense, as depicted by their low defect content. According to Zhong et al. [18], the optimization of fabrication parameters resulted in fully dense components and a reduced amount of defects. A stripe pattern scanning strategy was incorporated to ensure that the powder particles were fully melted, hence avoiding incomplete fusions. Vallejo et al. [19], identified that low volumetric energy density resulted in an increased lack of fusion defects, whereas high volumetric energy density resulted in the propensity for keyhole defects. These defects were detrimental to the mechanical properties, as they acted as sources of stress concentrations, leading to premature failures of the materials.

During the L-PBF process, the rapid cooling rates are typically high and range from 10^5^ to 10^8^ K/s, compared with conventional casting rates, which typically occur at much lower rates of 10^1^ to 10^3^ K/s [20,21,22]. The rapid heating and cooling process results in large thermal gradients that then control the resulting microstructure, grain size and shape, and grain orientation. Additionally, the thermal gradients result in the formation of either cellular, dendritic, or equiaxed subgrain features. The subgrain structures that form are usually finer compared with as-cast or as-forged stainless steels and contribute significantly to their mechanical properties [23].

The microstructures formed during additive manufacturing are significantly influenced by the size of the sample, primarily due to differences in heat dissipation rates [11]. Therefore, it is essential to consider the sample size when designing and optimizing the additive manufacturing process to achieve the desired microstructure. Leicht et al. [24] studied the microstructure of thin-walled parts, from 0.2 mm to 3 mm, and reported that thinner samples had an almost random texture, whereas thicker samples depicted grains oriented in the build direction. Niendorf et al. [25] studied the effect of diameter on the microstructures of SLM 316L stainless steel and reported that the thinner samples exhibited coarse and more elongated grains than the thicker samples. According to Roach et al. [26], the strength and stiffness of L-PBF 316L stainless steel specimens decreased dramatically as the specimen size reduced from 6.25 mm to 0.4 mm. The authors attributed the observed changes to the variation in surface roughness of the specimens. From these studies, it can be interpreted that the size of the sample plays a crucial role in the ultimate mechanical properties observed. It has been established that the processing parameters, such as laser power, play a significant role in the microstructures formed, and thus, the mechanical properties [27]. Most studies, however, did not clearly provide the outlined fabrication parameters used, which makes it a challenge to make a clear comparison between the properties observed.

Even though the properties and deformation behavior of L-PBF 316L stainless steel have been well discussed in the existing literature, there is still a wide disparity in data. Any slight changes made to the L-PBF fabrication parameters can significantly affect the grain size, texture, and even porosity. Therefore, the use of different fabrication parameters makes it impossible to make a conclusive comparison between one study and another. Therefore, with regard to previous studies, this study aimed to conduct an in-depth microstructural analysis of L-PBF 316L stainless steel. The fabrication parameters are clearly provided to aid other works in making a comparison with the results observed therein. Furthermore, different thicknesses are investigated with the goal of making a significant contribution to the crucial role played by part thicknesses. By understanding the size-dependent effects on the microstructure, tailored strategies can be developed to optimize the microstructural properties of various materials printed via L-PBF. The extensive insights obtained from this research are expected to make a meaningful impact on the ongoing research on additively manufactured materials and pave the way for further advancements.

## 2. Materials and Methods

The chemical composition of the 316L powder used in the L-PBF fabrication of the samples is shown in Table 1, where it is compared with the chemical composition requirements of ASTM 240/A240M [28]. The chemical composition of the powders was determined using a combination of inductively coupled plasma emission spectroscopy (ICAP-OES) and spark combustion analysis.

**Figure 1:**
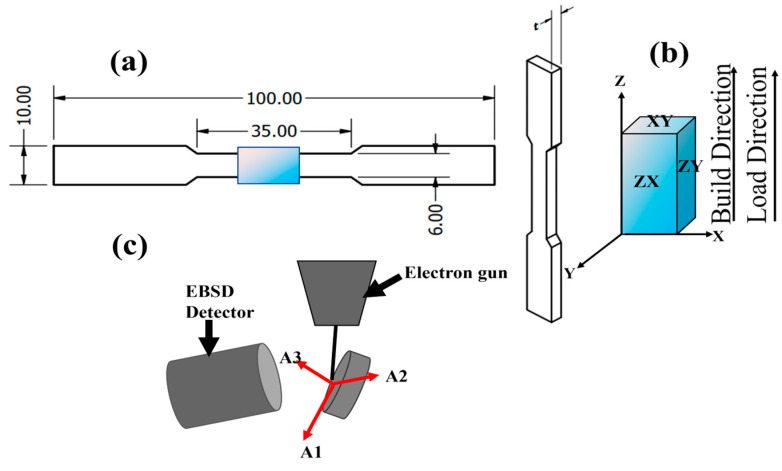
Schematic diagram of (**a**) the dog-bone-shaped samples illustrating the geometrical dimensions; (**b**) build direction and loading direction, with “t” indicating the thickness (1.5 mm and 4.0 mm); and (**c**) the scanning direction when observed under EBSD.

The 316L stainless steel dog-bone-shaped samples utilized in this study were provided by Honeywell Federal Manufacturing & Technologies (Honeywell FM&T), LLC, Kansas City, Missouri, and were manufactured using the L-PBF technique with Renishaw’s AM250 machine (Renishaw Engineering Company, Wotton-under-Edge, UK). The process parameters used to fabricate the samples were as follows: hatch power of 180 W, a hatch distance of 85 μm, a bulk hatch exposure time of 86 μs, and a layer thickness of 40 μm. A stripe scan strategy that involved bidirectional laser scanning without any rotation of the part or build platform was implemented. This strategy allowed for the fabrication of uniform melt pools, which is a crucial factor in ensuring the even layer-by-layer fabrication of the final part [29]. The printing parameters used are summarized in Table 2. The samples were fabricated vertically in the Z-direction, as illustrated in Figure 1. The fabrication was conducted under a nitrogen-inert atmosphere to minimize the oxidation of the samples. The fabricated samples were cleaned, and extra powder was removed from their surfaces via sandblasting. The samples were then removed from the mild carbon steel substrate using an electrical discharge machine.

The samples were fabricated in accordance with ASTM E8 specifications. The samples used in this study were of two different thicknesses (t) of 1.5 mm and 4.0 mm, whereas the other dimensions were kept constant.

Tensile tests were conducted using an Instron 5569A dual-column tabletop testing machine (manufactured by Norwood, Norfolk County, MA, USA) at room temperature under a strain rate of 0.001/s. An extensometer with a gauge length of 10 mm was used to measure the strain. The extensometer was removed halfway due to its limited range, and the tests were resumed until fracture. For each thickness, a minimum of five samples were tested, and the average results for the tensile properties obtained were reported. Hardness measurements were performed on the surface of the gauge sections of the samples using a Rockwell hardness tester (B-scale) (manufactured by Bridgewater, Somerset County, NJ, USA) following the ASTME18-20 specifications. A load of 100 kgf was applied for a dwell time of 10 s, and indents were made along a straight line. The average hardness measurement was obtained by taking measurements from a minimum of five samples. It is worth noting that the samples used in this study did not undergo any surface treatments, such as polishing, or post-fabrication processes, such as heat treatments.

The microstructures of L-PBF 316L stainless steel were investigated on both undeformed and deformed samples. The investigation of the as-built samples was considered necessary to assess the quality of the samples. The as-built microstructures would act as the baseline for comparison with the microstructures of the deformed samples. Optical Microscopy (OM) was used to observe the melt pools, scanning electron microscopy (SEM) equipped with an EBSD detector was used to observe the texture and grain orientation, and Electron Dispersive X-ray Spectroscopy (EDS) was used to determine the chemical composition of the samples. To prepare the samples for the OM and EBSD characterization, they were initially sectioned along the ZX plane using a conventional band saw. The faces of the sectioned samples were cold mounted and then mechanically polished using silicon carbide paper of different grit sizes of 320, 600, 800, and 1200 grit. Water was used to remove any abrasive materials from the surfaces of the samples before changing to the next grit size. Subsequently, the samples were cloth polished using diamond pastes of 3 μm and 1 μm before being finally polished using 0.05 μm colloidal silica to obtain a mirror finish. The samples were then cleaned ultrasonically with ethanol and dried under cool airflow. The polished unetched surfaces were observed under a Nikon Eclipse MA200 OM (manufactured by Nikon Instruments Inc., Melville, NY, USA) to determine the porosity of the materials with the aid of ImageJ software (Java 1.8.0_172). The mirror finish samples were then etched using Kalling’s reagent (obtained from Pace Technologies, Tucson, AZ) for 40 to 60 s to reveal the microstructure. The etched samples were observed using OM and SEM. The EBSD technique was also used to observe the etched surfaces by considering a scan area of 200 × 200 μm^2^ and a step size of 1 μm at an acceleration voltage of 20 kV. The post-processing of the EBSD data was carried out using orientation imaging microscopy (OIM) analysis software version 7 to obtain inverse pole figure (IPF) maps, grain boundary maps, and kernel average misorientation (KAM) maps.

## 3. Results

### 3.1. Microstructural Characterization of Undeformed Samples

Table 3 summarizes the results of the elemental composition analysis of the samples conducted using EDS. High amounts of nitrogen were observed and were attributed to the nitrogen-inert atmosphere used in fabricating the samples. It is important to note that EDS can be restricted by its resolution and might not measure down to the nano level. However, the variations in the chemical composition of the target areas can still give a relative element amount of the base material.

Porosity remains a main concern in additive manufacturing. It affects the load-bearing capabilities of the materials, thus lowering their strengths [30]. With the aid of ImageJ software (Java 1.8.0_172) the porosity areal distribution, average size, and size distribution were determined. Based on this investigation, an average of 0.05% and 0.03% porosity for the 1.5 mm and 4.0 mm thick samples, respectively, were determined. The lower porosities observed for both thicknesses are indicative of relatively high densities of the fabricated samples. The slight variation in the porosities of the two thicknesses implied that there was a potential change in the porosity as the thickness changed. The change in porosity with thicknesses might have been caused by the different thermal gradients, as thicker samples are likely to dissipate heat faster than thinner samples. Further tests using the Archimedes method will be considered in future works to confirm these findings.

In a previous study by Sarafan et al. [31], it was demonstrated that the porosity of samples could be influenced by the laser power. Increasing the laser power from 160 W to 380 W resulted in a corresponding increase in density from 98.1% to 99.5%. Therefore, based on the results obtained in this study, it can be inferred that the 180 W laser power used in combination with other processing parameters significantly contributed to the relatively low porosities observed.

**Figure 2:**
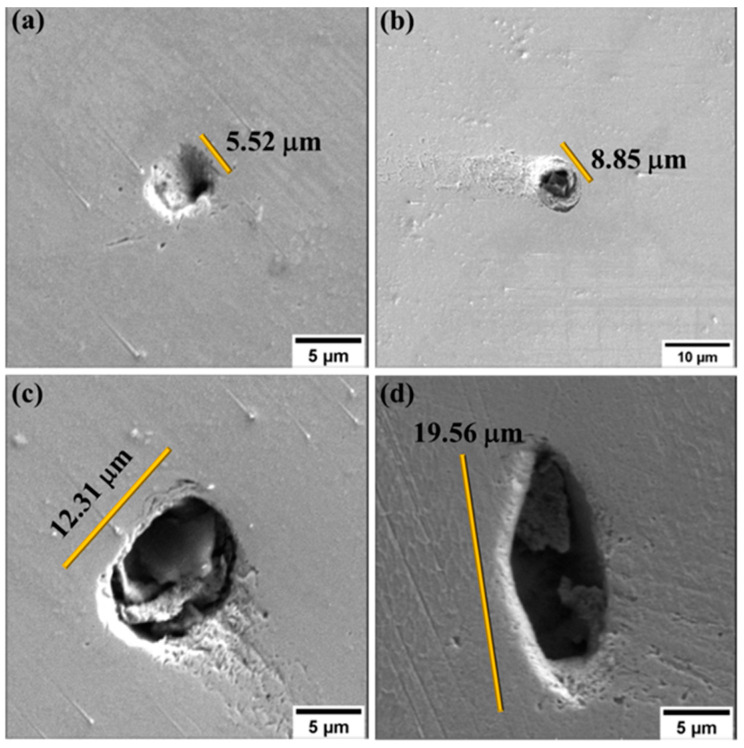
SEM images at higher magnification where (**a**,**c**) represent 1.5 mm and (**b**,**d**) represent 4.0 mm, showing pores with different sizes and shapes and with unmolten materials inside.

An in-depth examination of the polished undeformed surfaces was conducted using SEM. These images were obtained to understand the factors contributing to the observed porosities. It was observed that the pores occurred randomly across the surface of the samples. Figure 2 illustrates the presence of unmolten materials inside the pores. The pores were observed to be of different sizes and shapes. Even though these pores were observed on the surfaces of both thicknesses, low percentages of porosities were still recorded.

**Figure 3:**
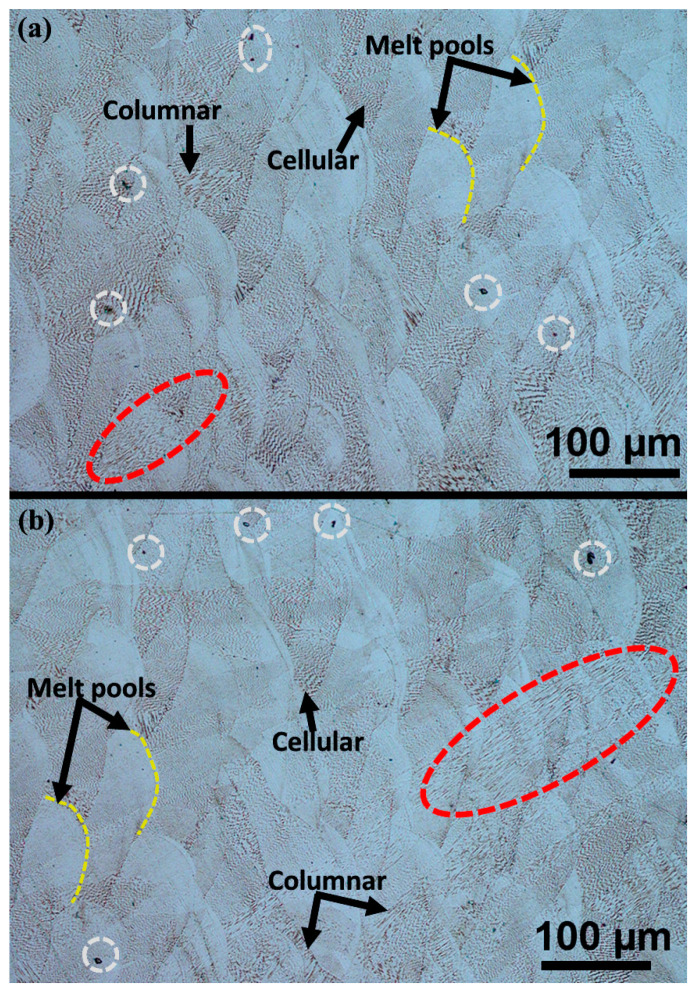
Optical micrographs of etched surfaces observed at 20× magnification for (**a**) 1.5 mm and (**b**) 4.0 mm. The white circles indicate dark spots at the melt pool boundaries, the red dotted circles indicate epitaxial growth, and the yellow dots indicate melt pools.

The samples etched with Kalling’s reagent revealed the presence of melt pools, as shown in Figure 3a,b. These melt pools exhibited an arc shape, which resulted from the layer-by-layer deposition process employed in additive manufacturing. As the new layers were deposited on top of previously deposited layers, the laser beam partially remelted the previous layer, leading to the observed overlaps. The rapid solidification rates of L-PBF, usually in the range of 10^5^ to 10^8^ K/s, resulted in the formation of fine cellular structures and columnar dendritic structures [17,23]. The temperature gradient G and the growth rate R play an important role in determining the type of structures formed during solidification. The G/R ratio is used to determine the solidification mode, while the product of G and R influences the size of the structures formed. At high G/R planar solidification features are formed, whereas at low G/R, cellular or dendritic features are formed. When G × R is high, finer and more uniform structures are formed. Therefore, it is crucial to control the temperature gradient and growth rate in the L-PBF process to ensure the desired solidification features, which can impact the mechanical properties, are obtained [32,33]. Epitaxial growth, where the cellular structures grow across melt pool boundaries while retaining their preferred growth direction [1], is depicted by the red dotted circles. Dark spots were observed on the etched surfaces, as depicted by the white dotted circles. These dark spots were observed to mainly occur at the melt pool boundaries and were assumed to be incomplete fusions or pores due to temperature gradients.

**Figure 4:**
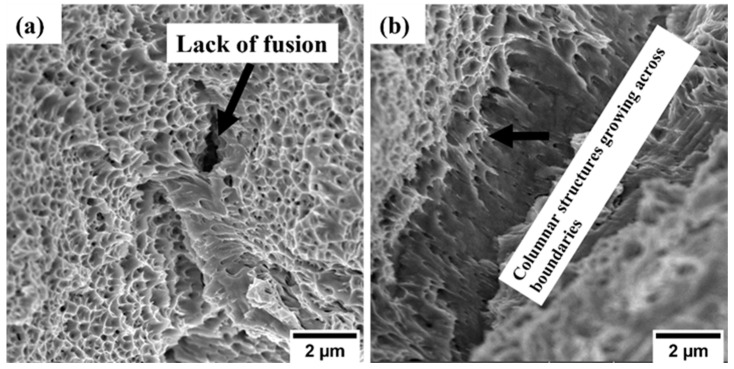
SEM images illustrating cellular and columnar structures: (**a**) 1.5 mm and (**b**) 4.0 mm.

A higher magnification of these surfaces showed the formation of finer structures at sub-micron levels, as shown in Figure 4a,b. A lack of fusion was observed in the 1.5 mm sample, which occurred when the solidification process occurred rapidly, causing incomplete fusion of the melt pools [34]. The temperature gradients between layers could result in different morphologies, with high temperatures resulting in columnar structures growing across the melt pool boundaries, as depicted in Figure 4a,b.

**Figure 5:**
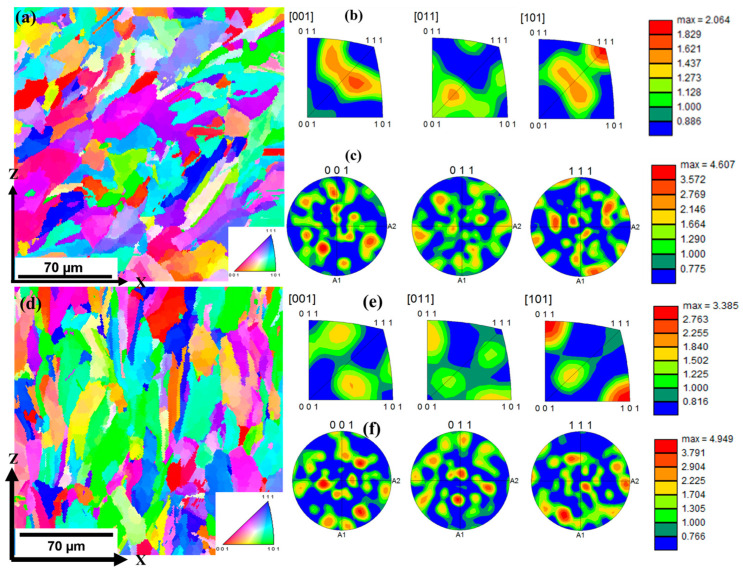
IPF maps of undeformed L-PBF 316L stainless steel with their respective inverse pole figure plots and pole figures. (**a**–**c**) represent 1.5 mm thick samples, whereas (**d**–**f**) represent 4.0 mm thick samples.

The as-built microstructures were further characterized using EBSD to understand their grain morphologies. Figure 5 shows the IPF maps with their respective inverse pole figure plots (IPFPs) and pole figures (PFs) for both thicknesses. The pole figures were used to obtain the crystallographic orientation of the grains with respect to the scanning plane. The IPFPs, on the other hand, were used to obtain the crystallographic orientation of individual grains with respect to the scanning direction. From the IPFP figures, it can be inferred that the samples had a preferred crystallographic orientation of <101> toward the build direction (*Z*-axis). The 1.5 mm thick sample showed a slight tilt in the orientation of the grains from the build direction, whereas the grains in the 4.0 mm thick sample grew parallel to the build direction. The slight tilt may have resulted in the reduced relative frequency of grains oriented in the <101> direction, as indicated by the intensity bar of 1.5 mm compared with that of 4.0 mm. The pole figures showed that the grains were oriented normal to the {001} plane. Thus, a texture of {001}<101> was observed for both thicknesses. Other studies have also observed the preferred crystallographic orientation along the <101> direction [35,36].

**Figure 6:**
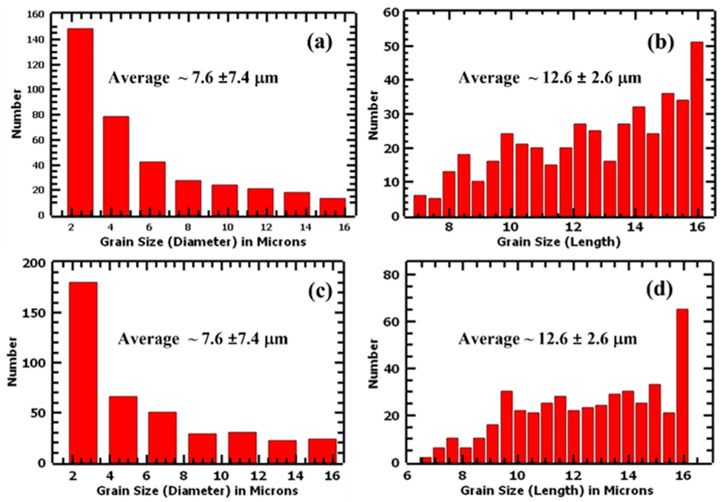
Average grain size distribution charts (diameter and length) of undeformed L-PBF 316L stainless steel samples: (**a**,**b**) are 1.5 mm and (**c**,**d**) are 4.0 mm.

Columnar grains were observed in all samples, and the preferred orientation is represented by the intensity bars. The grains were observed to be of different sizes, ranging from hundreds of nanometers to tens of microns lengthwise. The average grain size distributions (diameters) were measured to be 7.6 ± 7.5 μm and 7.6 ± 7.4 μm for the 1.5 mm and 4.0 mm samples, respectively. The average grain lengths were determined to be 12.6 ± 2.6 μm and 12.6 ± 2.6 μm for 1.5 mm and 4.0 mm, respectively. Figure 6 shows the grain size distributions of the undeformed samples. Based on these results, it can be concluded that there was no significant difference in the grain sizes of the two thicknesses in their as-built states. Research showed that additive manufacturing produces finer grains compared with conventional manufacturing methods. For instance, Bartolomeu et al. [23] determined the average grain size of cast 316L stainless steel to be 91 ± 17 μm and those of SLM 316L stainless steel as 13 ± 4 μm. Similarly, Jaskari et al. [37] obtained an average grain size of 62 μm for wrought 316L and an average grain size ranging from 10.5 to 15.3 μm for SLM 316L stainless steels. The fine grains contribute significantly to the mechanical properties of L-PBF samples as per the Hall–Petch relationship [38]. However, as explained in the discussion section, the predominant strengthening mechanism was observed to originate from cellular structures rather than primarily from grain sizes.

**Figure 7:**
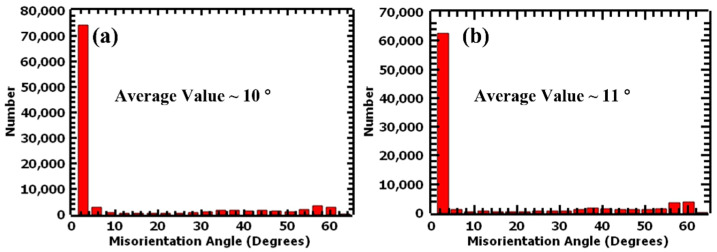
Average misorientation angle distribution charts of undeformed L-PBF 316L stainless steel: (**a**) 1.5 mm and (**b**) 4.0 mm.

The average misorientation angle of the grains was also determined to be 10° and 11° for the 1.5 mm and 4.0 mm samples, respectively. Both thicknesses demonstrated low misorientation of the grains in their as-built state. The one-degree difference between the two thicknesses did not significantly affect the orientation of the grains and was confirmed by the same texture {001}<101> for both thicknesses. Figure 7 shows the average misorientation angle distribution charts for the as-built samples.

**Figure 8:**
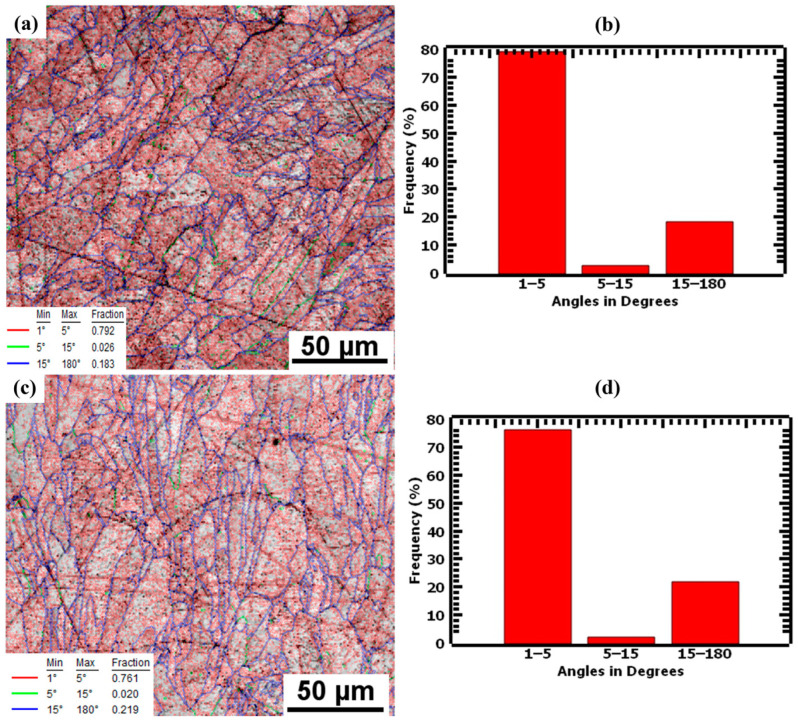
Grain boundary maps of undeformed samples showing LAGBs marked by red and green lines and HAGBs marked by blue lines for (**a**) 1.5 mm and (**c**) 4.0 mm, and (**b**,**d**) as their repective distribution charts.

Grain boundary maps were used to investigate the low-angle grain boundaries (LAGBs) and high-angle grain boundaries (HAGBs) of as-built samples. Grain boundaries ≤15° were considered LAGBs, whereas those ≥15° were considered as HAGBs. The grain boundary maps in Figure 8 show that as-built L-PBF samples have a large fraction of LAGBs. The 1.5 mm samples had 81.8% LAGBs and 18.3% HAGBs, whereas the 4.0 mm samples had an average of 78.1% LAGBs and 21.9% HAGBs. The high percentages of LAGBs and the low misorientation angles of the two thicknesses indicate that numerous sub-grain features, such as cellular structures, existed in the microstructure. These cellular structures have cell walls that act as strengthening mechanisms by hindering dislocation motion [39].

### 3.2. Mechanical Properties

**Figure 9:**
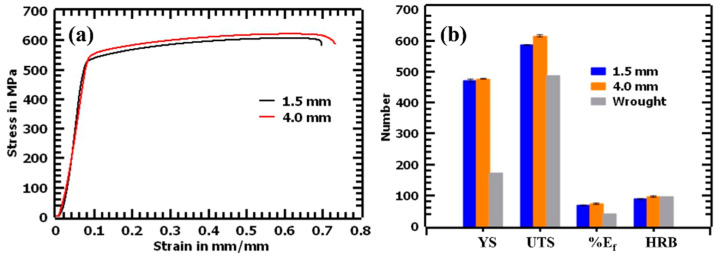
(**a**) Stress–strain curves of 1.5 mm and 4.0 mm thick samples and (**b**) average YS, UTS, E_f,_ and HRB values for L-PBF and ASTM standard values.

Figure 9a shows the stress–strain curves of the tested L-PBF 316L stainless steel samples. Figure 9b shows a chart comparing the YS, the UTS, the elongation to fracture (E_f_ (%)), and the hardness (HRB) of the tested materials, along with their corresponding values for the standard ASTM A240/A240M-18 wrought steel [28]. The 4.0 mm thick sample exhibited slightly better tensile properties than the 1.5 mm thick sample. The UTS value was higher by 2.4% (619.58 ± 3.23 MPa), YS by 2.1% (551.21 ± 2.43 MPa), elongation to failure by 5.8% (73.66 ± 3.02%), and hardness value by 6.9% (93 ± 1.5 HRB), compared with the 1.5 mm thick sample. The mechanical properties demonstrated low scatter in data, which is attributed to the fully dense properties of both thicknesses. These results are summarized in Table 4.

Young’s modulus was determined to be 156.52 GPa and 156.63 GPa for the 1.5 mm and 4.0 mm thick samples, respectively. Young’s modulus seemed to be less influenced by the thickness of the samples, as depicted by the minimal variation in the values of the two thicknesses. The values observed were lower than those of the ASTM standards, where a value of 193 GPa is usually expected [28]. Even so, the values obtained were comparable with those in the literature, where Young’s modulus of 142 to 182 GPa was measured for vertically built samples [40]. The average hardness value of the L-PBF samples was determined to be 87 ± 0.5 HRB for the 1.5 mm thick samples and 93 ± 1.5 HRB for the 4.0 mm thick samples. The ASTM 240/240M-20a standards specify that the maximum hardness of 316L stainless steel manufactured through casting or forging is 95 HRB, with most conventionally manufactured materials exhibiting hardness values much lower than 95 HRB. Due to the fact that the standard specifications for additively manufactured materials are still being developed, this study used Renishaw datasheet values as a basis for comparison [41]. The Renishaw data sheet specifies a typical hardness value of 95 HRB. Thus, the results obtained from both thicknesses in this study were close to the stipulated value.

Conventionally manufactured materials usually report that an increase in YS is accompanied by a decrease in ductility [42]. High strengths and high ductility are usually observed for twinning-induced plasticity (TWIP) steels [43,44]. However, in this study, the YS and ductility of L-PBF 316L stainless steel samples increased simultaneously without a tradeoff. Furthermore, the hardness values increased irrespective of the samples not being polished. These features were attributed to the unique microstructures formed, and thus, the need to observe the deformed surfaces of the L-PBF samples.

### 3.3. Microstructural Characterization of the Deformed Samples

**Figure 10:**
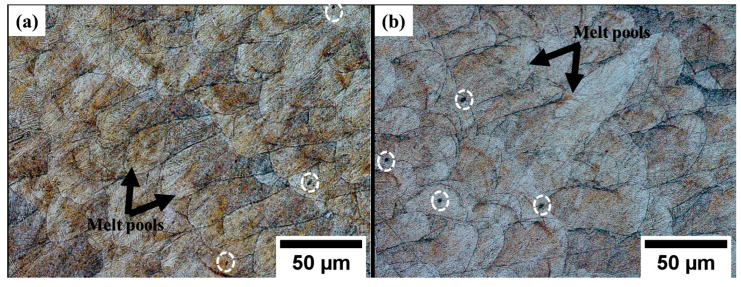
Optical micrographs of deformed etched samples showing enlarged and elongated melt pools and the white dotted circles showing dark spots representing pores: (**a**) 1.5 mm and (**b**) 4.0 mm.

The deformation mechanisms of the L-PBF 316L stainless steel samples were investigated using OM, SEM, and EBSD. Etching of the cut and polished ZX cross-sections of the samples revealed the melt pools shown in Figure 10. The melt pools appeared elongated toward the tensile load direction and enlarged compared with those of as-built surfaces. Some melt pools appeared more elongated compared with others. This could have been due to the difference in the crystal orientation relative to the applied force. The grains with a more favorable crystallographic orientation for dislocation were likely to deform easily, leading to a higher degree of elongation [45]. A few dark spots marked with white dotted circles were identified as pores. These pores were observed to be randomly distributed on the fractured surfaces, with most of them occurring at or close to the melt pool boundaries.

**Figure 11:**
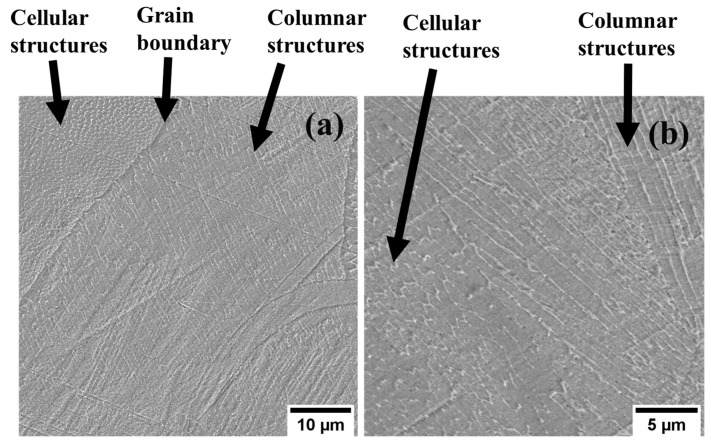
Representative SEM images of the etched L-PBF 316L stainless steel samples showing cellular and columnar structures: (**a**) 1.5 mm and (**b**) 4.0 mm.

SEM images of the deformed surfaces were also obtained. Figure 11 reveals that deformation occurred at the sub-micron level, as illustrated by the elongated cellular and columnar structures.

**Figure 12:**
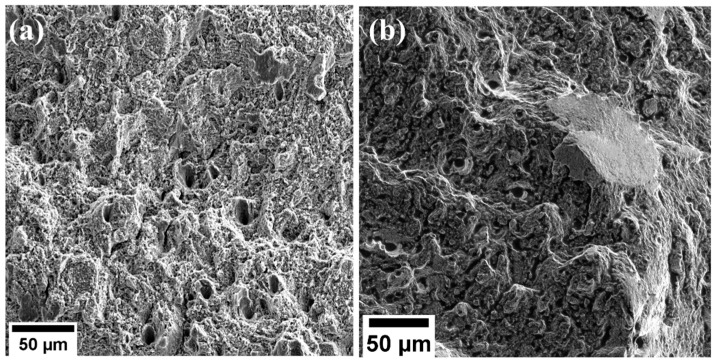
SEM fractography of tensile-tested samples, illustrating ductile fracture mode: (**a**) 1.5 mm and (**b**) 4.0 mm.

Figure 12 shows a higher magnification of the surfaces. Numerous dimples were observed on the surfaces, illustrating a ductile fracture mode.

**Figure 13:**
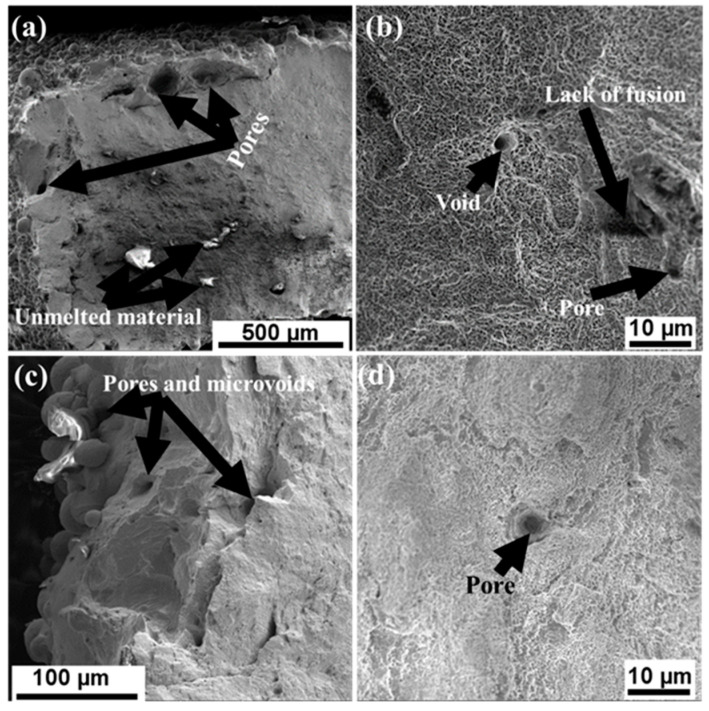
SEM images of L-PBF 316L stainless steel samples depicting numerous defects: (**a**,**b**) represent 1.5 mm, whereas (**c**,**d**) represent 4.0 mm.

Several defects, including lack of fusion, pores, voids, unmelted materials, and microcracks, were identified on the fractured surfaces, as illustrated in Figure 13. It is worth noting that the as-built microstructures exhibited fewer defects compared with the deformed samples. This difference can be ascribed to the initial polishing of the as-built microstructures before the examination, which was a step that likely eliminated any residual unmelted particles or defects present on the surfaces.

Moreover, in the course of deformation, microvoids were initiated at regions characterized by localized strain discontinuities. Pre-existing defects, such as pores and lack of fusions, observed in the as-built undeformed surfaces served as favorable sites for the nucleation or growth of microvoids. With increasing tension loads, the pores merged to form microvoids, and these microvoids expanded and coalesced with adjacent ones. This resulted in the numerous voids observed on the fractured surfaces of deformed samples compared with the undeformed samples. The closer the pre-existing pores were to each other, the faster they promoted the nucleation and merging of microvoids [46], as shown in Figure 13. The presence of unmelted particles shown in Figure 13a,c were formed due to the laser power insufficiently melting the powder particles. Remelting of the samples can aid in reducing the number of unmelted particles during fabrication [47]. The formation of these defects at the edges causes premature failure of the material.

**Figure 14:**
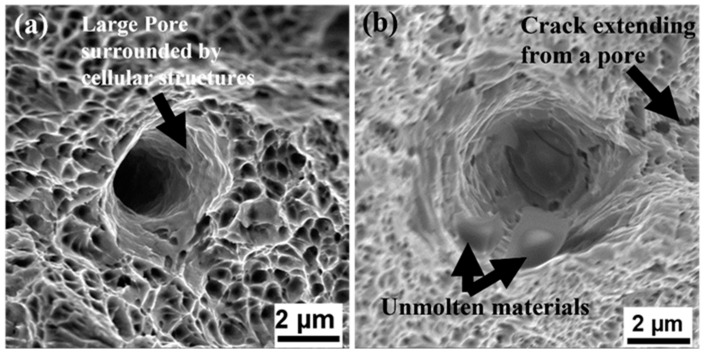
SEM fractographs showing defects formed at nano-level: (**a**) 1.5 mm and (**b**) 4.0 mm.

Furthermore, the high-stress concentration near the defects can lead to the nucleation and propagation of cracks that can ultimately lead to the failure of the material. Figure 14 shows the presence of microcracks nucleating from or close to the pores.

**Figure 15:**
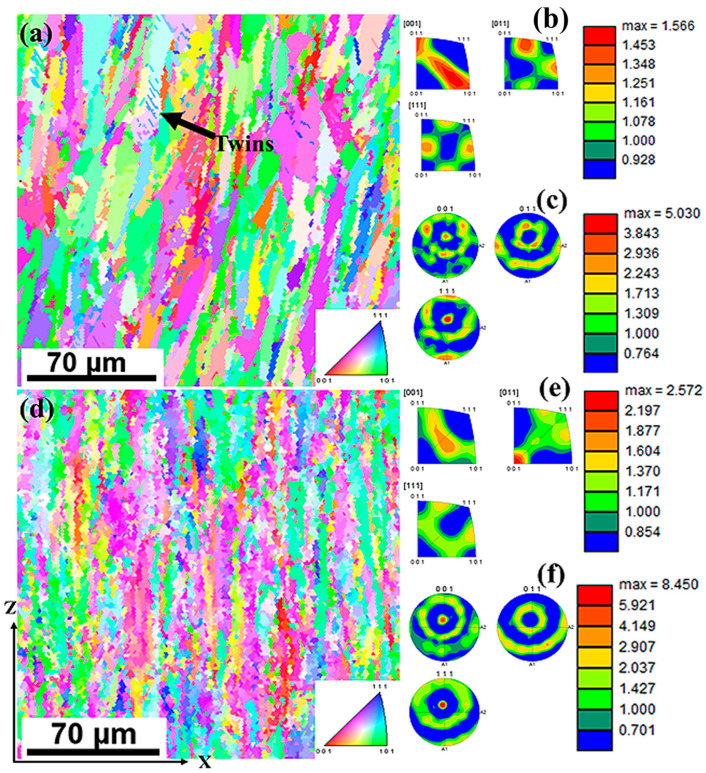
IPF maps of deformed L-PBF 316L stainless steel with their respective inverse pole figure plots and pole figures: (**a**–**c**) represent 1.5 mm and (**d**–**f**) represent 4.0 mm thick samples. Grains had a preferred orientation toward the *Z*-axis in both thicknesses.

EBSD analysis was used to supplement the SEM observation of fractured surfaces. Figure 15 shows the IPF maps of the deformed samples with a stereographic triangle and corresponding inverse pole figure plots and pole figures.

The 1.5 mm sample was found to have a preferred crystallographic orientation along the <001> direction, while the 4.0 mm thick sample had a preferred orientation along the <011> direction. These orientations were different from the <101> orientation observed in the undeformed samples, suggesting that the deformation process had induced a change in the crystallographic orientation of the material. Moreover, the 1.5 mm thick sample had all the grains parallel to the build direction, unlike in its undeformed state, where the grains were slightly tilted. Wang et al. [34] demonstrated that the preferred orientation changed from <110> to <100> as the diameter decreased. Similar observations were made for the deformed samples in this study, where the thickness resulted in different preferred crystallographic orientations. The pole figures also showed a change in the preferred plane normal to the scanned surface from {001} to {111} for both thicknesses. The results from the IPFPs and PFs, therefore, suggest that the deformed 1.5 mm thick samples had a {111}<001> texture, whereas the 4.0 mm thick samples had {111}<011> textures.

Both thicknesses showed competition regarding the preferred crystallographic orientations between the <001> and <011> orientations. Specifically, the 1.5 mm sample had a stronger crystallographic orientation in <001> with a value of 1.566, followed closely by <011> with a value of 1.461. The 4.0 mm sample, on the other hand, had a stronger crystallographic orientation in <011> with a value of 2.572, followed closely by <001> with a value of 1.912. Since these values were quite close, further studies need to be conducted on a wider range of scales to establish the impact of thickness on the crystallographic orientation of L-PBF 316L stainless steel. The slight differences in the tensile properties between the two thicknesses could also be attributed to these observed slight differences in the crystallographic orientation and intensities. Thus, it can be inferred that in this study, the grains with the <011> orientation were more favorable for plastic deformation and dislocation motion, leading to higher ductility and elongation under tensile loading [45]. Based on literature works [34,36] and this study, it is clear that the crystallographic orientation of the samples is geometry dependent, as well as parameter dependent. Therefore, the properties can be tailored to achieve the desired properties.

**Figure 16:**
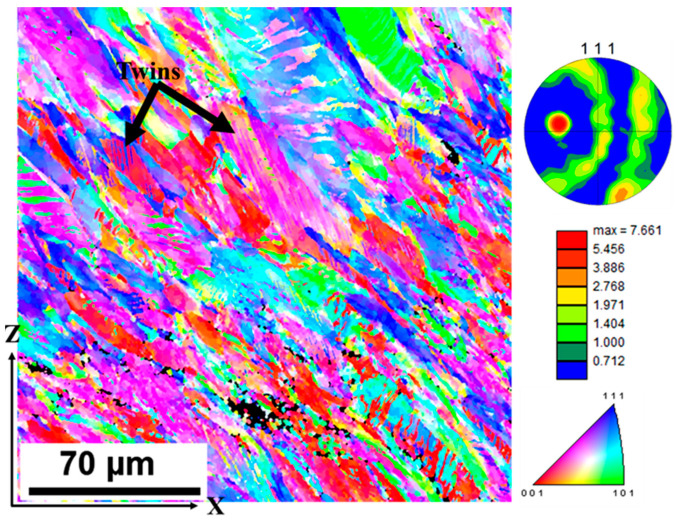
Dominant deformation twinning observed at a 0.3 μm step size.

A closer observation of the IPF map in Figure 15a shows the existence of twins. To further clarify these occurrences, the surface was observed at a smaller step size of 0.3 μm. The EBSD step size was reduced because the step size determines the size of grains observed. With a step size of 1 μm, grains or features smaller than 1 μm can be filtered out. Thus, at 0.3 μm step size, twinning was quite evident in the deformed surface. Twinning, unlike slip, is polar in nature and relies strongly on the size of the grains and the strain rate [43]. Sinha et al. [48] illustrated that at room temperature, damage accumulates along the <100>-oriented grains, while the <101>-oriented grains enhance the strain hardening of the materials through twinning. Thus, the <101> grains observed in the undeformed state can be attributed to the enhanced strength and the induced deformation twins. The deformation mode of twinning was previously observed in the EBSD analysis of additively manufactured 316L stainless steel samples [49,50]. The {111} plane, which is usually closely packed in face-centered-cubic (fcc) crystal materials, is suitable for easy dislocation gliding and consequently promotes twinning [45]. Figure 16 shows that twinning mostly occurred in the {111}-oriented grains. The observed twins were found to be closely spaced.

Additionally, the high yield stress reached by the L-PBF 316L samples encourages the early development of deformation twinning. When a high yield stress is reached, the deformation becomes more localized and concentrated in specific regions, such as the grain boundaries [51]. Deformation twinning becomes preferred over slip because it allows for the accommodation of the plastic deformation with minimal energy consumption by producing a strain field with an opposite sign to the applied stress. The occurrence of deformation twinning reduces the mean free path of dislocations, consequently leading to enhanced strength and strain hardening of the material [43].

**Figure 17:**
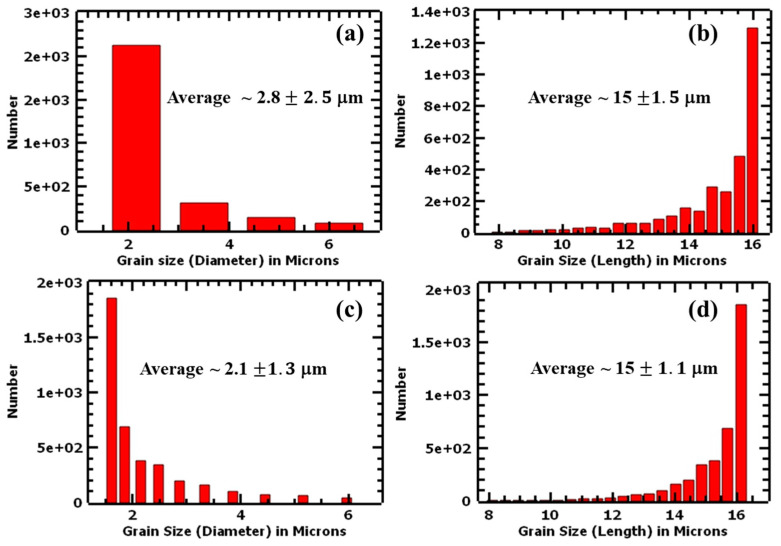
Average grain size, where (**a**,**b**) represent the diameters and lengths of 1.5 mm thick samples and (**c**,**d**) represent the diameters and lengths of 4.0 mm thick samples.

The IPF maps show columnar grains that were more slender than those of as-built IPF maps and were oriented toward the build direction. The results of the average grain sizes of the deformed samples are summarized in Figure 17. The average grain size distributions (diameters) for the deformed samples were 2.8 ± 2.5 μm for the 1.5 mm sample and 2.1 ± 1.3 μm for the 4.0 mm sample. The grain size diameter was thus observed to have reduced compared with the values obtained from the undeformed samples. The average grain size lengths of the samples were determined to be 15 ± 1.5 μm and 15 ± 1.1 μm for the 1.5 mm and 4.0 mm thick samples, respectively, and it was confirmed that the grains incurred elongation due to tensile loads. Similar to the undeformed samples, the two thicknesses, in their deformed state, did not show a significant difference in their average grain sizes.

**Figure 18:**
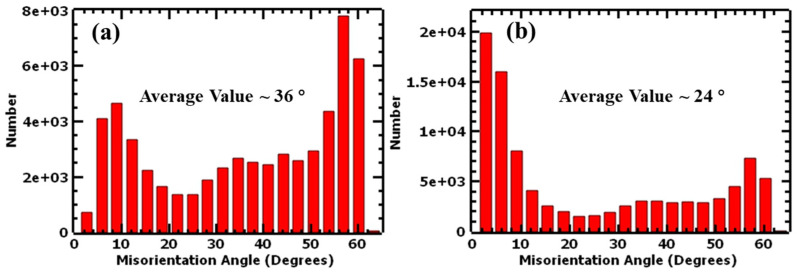
Average misorientation angles of deformed (**a**) 1.5 mm and (**b**) 4.0 mm thick samples.

The misorientation of grains was also determined for the two thicknesses. It was observed that the grains in the 1.5 mm thick sample experienced higher misorientation than those of the 4.0 mm thick sample, as shown in Figure 18. Additionally, the deformed samples exhibited a wider distribution of grain misorientation compared with the sharp peaks observed at smaller angles in the undeformed samples. When the samples were subjected to tensile loads, lattice distortion of the crystals occurred. Furthermore, the migration of pre-existing grain boundaries caused a change in the orientation of grains with respect to the adjacent grains. The change in crystallographic orientations led to higher misorientation angles between them.

**Figure 19:**
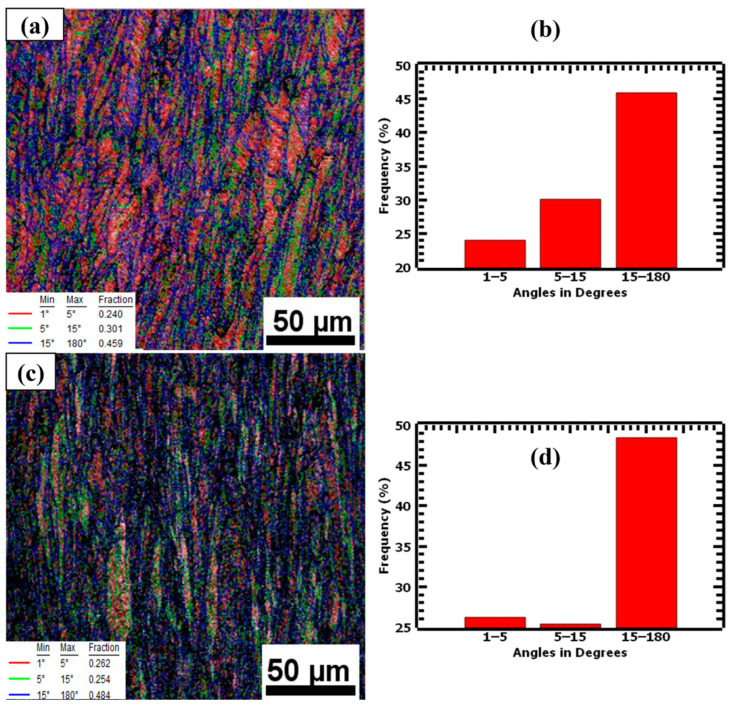
Grain boundary maps of deformed (**a**) 1.5 mm and (**c**) 4.0 mm L-PBF 316L stainless steel samples, and (**b**,**d**) as their respective distribution charts. The grain boundary maps show the HAGBs in blue color and LAGBs in red and green colors.

The plastic deformation of the samples resulted in the lattice distortion of crystals leading to the misorientation of grains and eventually the formation of HAGBs [52]. Furthermore, the weaker resistance to deformation by the LAGBs caused them to migrate and combine with grain boundaries with similar orientations to form HAGBs. The evolution of grain boundaries due to plastic deformation was evaluated using grain boundary maps, as shown in Figure 19. The deformed samples demonstrated a significant increase in the number of HAGBs, with an increase from 21.9% to 45.9% for 1.5 mm, and from 18.3% to 48.4% for 4.0 mm. The HAGBs formed had higher resistance to dislocation motion. As a result, higher stress was required to cause dislocation motion, leading to strain hardening of the material. During the strain-hardening stage, dislocations accumulated at HAGBs and generated higher stress concentrations. The increased interaction and entanglement of dislocations enhanced the materials’ strength [16]. Therefore, the grain boundaries, fine grains, and cellular structures collectively contributed to the enhanced strength and ductility of the L-PBF 316L stainless steel samples.

**Figure 20:**
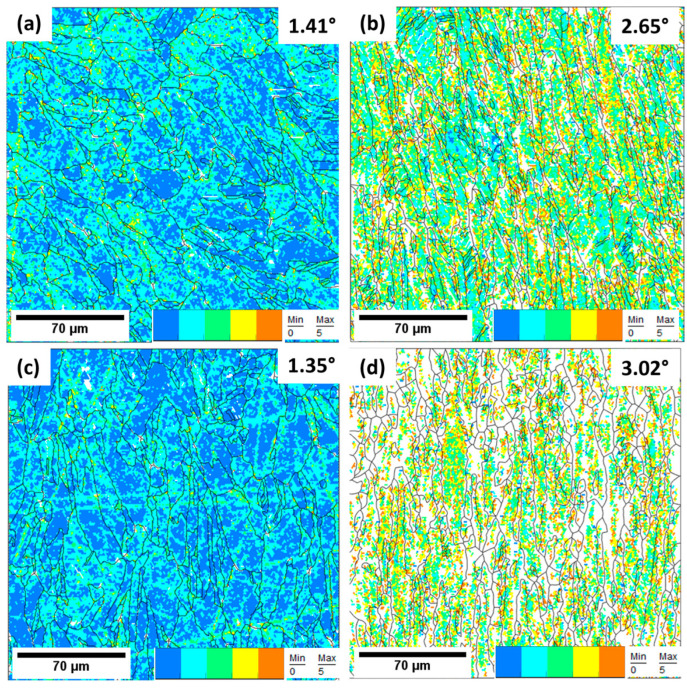
KAM maps of L-PBF 316L stainless steel with (**a**,**c**) representing undeformed samples and (**b**,**d**) representing deformed samples. (**a**,**b**) represent 1.5 mm, whereas (**c**,**d**) represent 4.0 mm. The intensity of localized strain was observed to increase when the samples were subjected to tensile loads, as illustrated by the increased KAM values.

Kernel average misorientation (KAM) maps were used to represent the average misorientation of a given point (kernel) with respect to that of its neighbors in the same grain The KAM maps were used to provide additional information on the lattice distortions and the deformation localizations [53]. The first nearest neighbor was used, and a misorientation angle lower than a threshold of 5° was selected to exclude the influence of other features, such as the grain boundaries [54]. The KAM maps in Figure 20 represent the undeformed and deformed samples. The scale bar illustrates the KAM values, with blue indicating less than 1°, turquoise indicating values ranging from 1° to 2°, green from 2° to 3°, yellow from 3° to 4°, and orange from 4° to 5°. The blue in the KAM maps represents low dislocation densities, while the orange spots indicate regions with higher dislocation densities.

As observed in the KAM maps, the undeformed samples were mainly dominated by the blue color, indicating less lattice distortion. Yellow and orange spots were observed on these surfaces as well and could be attributed to residual stresses that developed during the rapid solidification process [13,37,55]. When subjected to the tensile loads, lattice distortion occurred, and thus, the change in color from blue to green and yellow. Additionally, the dislocations piled up at the grain boundaries, leading to highly localized strain at these sites, as depicted by the orange colors. The 4.0 mm thick samples had higher KAM values than the 1.5 mm thick samples, demonstrating higher resistance to dislocation motion. These results support the observed mechanical properties of the 4.0 mm thick samples, where higher tensile strengths than those of 1.5 mm samples were observed.

**Figure 21:**
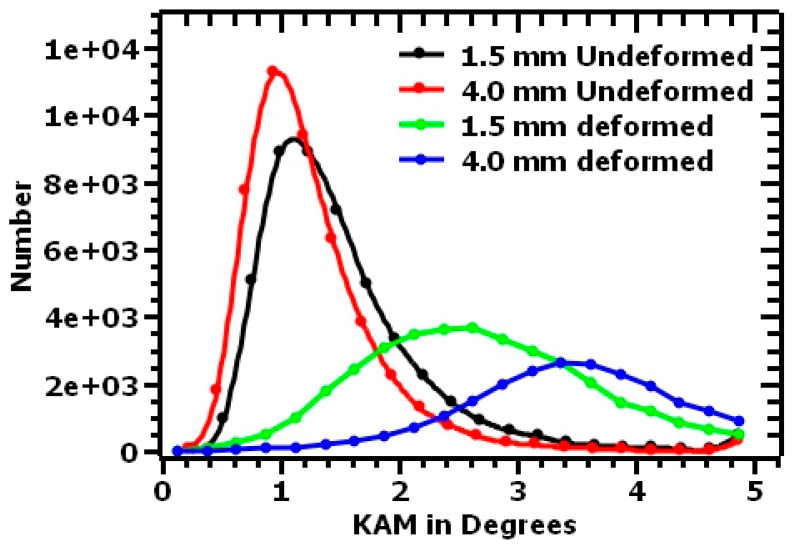
Kernel average misorientation angle distribution charts for undeformed and deformed L-PBF 316L stainless steel.

KAM distribution charts obtained from the KAM maps are shown in Figure 21. The KAM charts indicated the differences in the distribution of the misorientations between the undeformed and the deformed samples. The undeformed samples were observed to have a sharp peak in the lower misorientation angles, around 1°, with the 4.0 mm thick samples having a sharper peak before flattening out at higher misorientation angles. On the other hand, the deformed samples demonstrated a shift in the peak from lower misorientation angles to higher misorientation angles. The stress accumulation due to plastic deformation, which was attributed to the presence and rearrangement of dislocations, resulted in the observed increased KAM values of deformed samples.

## 4. Discussion

As shown in Table 4, the mechanical properties of the L-PBF 316L stainless steels were higher than the ASTM 240/A240M standard specification values for conventionally manufactured materials. The microstructural analysis of the L-PBF 316L stainless steel revealed unique features formed by the rapid solidification process. The cellular structures observed were at the submicron scales and were identified to contribute to the enhanced YS of additively manufactured materials. These cellular structures had cell sizes ranging from 0.2 to 1.0 μm. Theoretically, the Hall–Petch relationship states that higher material YS can be achieved by reducing the grain size [56]. The theory demonstrates the relationship between grain size *d* and the YS $\sigma_{y}$ of the material, as described by Equation (1):

(1) $$\sigma_{y}=\sigma_{o}+\frac{K_{\mathrm{HP}}}{\surd d}$$

where $\sigma_{o}$ is the Peierls stress for dislocation motion and $K_{HP}$ is the Hall–Petch constant for the material.

Therefore, using the average size from the cell sizes, namely, ~0.6 μm, considering that the Hall–Petch strengthening behavior is from the cell sizes, and using the Hall–Petch fitting plot obtained from [14,38,57], then

(2) $$\sigma_{y}=183.31+\frac{253.66}{\surd d}\left(\mathrm{MPa}\right)\left(d\;\mathrm{in}\;\mu m\right)$$

which gives an approximate YS of 510 MPa. This value is lower than the experimental values measured for the 1.5 mm and 4.0 mm thick samples, where yield strengths of 538 MPa and 551 MPa were measured, respectively. Suppose the grain size measured from the IPF maps was considered, where an average of 7.6 ± 7.5 μm and 7.6 ± 7.4 μm for the 1.5 mm and 4.0 mm, respectively, were determined; then, the approximate yield strengths will be 275.02 MPa and 275.20 MPa. These values are much lower than the experimental values measured. Thus, the values from the cellular structure sizes accounted for a larger portion of the yield strengths measured, demonstrating their high contribution to strengthening the materials produced via L-PBF. The remaining percentage of yield strengths could be attributed to other features, such as grain boundaries that hinder dislocation motion.

In fcc structures, plasticity occurs through the motion of dislocations, mainly through 12 potential {111}<110> slip systems [58]. As plastic deformation occurs, dislocation–dislocation interactions and dislocation–boundary interactions occur and substantially affect the strength and ductility of the materials. Dislocations can either be transmitted through gliding motions or be blocked by cell wall boundaries or grain boundaries [45]. By blocking the free movement of the dislocations, an increase in the applied stress is necessary for further plastic deformation to occur, leading to strain hardening. The result is the improved mechanical properties of L-PBF 316L stainless steel samples, including high strength.

Generally, it is known that the dislocations in a polycrystalline material can either be geometrically necessary dislocations (GNDs) or statistically stored dislocations (SSDs) [55]. Localized shearing and lattice rotation occurs during plastic deformation, causing grain misorientations [59]. Intergranular variations in the microstructure occur due to variations in dislocation densities and distributions. As a result, strain incompatibilities are formed in grains and grain boundaries. To achieve strain compatibility, the dislocations are trapped and stored, with the aim of minimizing the energy per unit length of dislocations. These dislocations are deemed necessary for maintaining strain compatibility, which gives rise to them being referred to as geometrically necessary dislocations [60]. These dislocations have a net non-zero Burgers vector and generate local lattice curvature or misorientation. The energy minimization achieved through the GNDs causes the formation of two-dimensional dislocation arrays (cellular dislocation walls). The grains are thus subdivided into subgrains. The newly formed boundaries divide the regions into cellular blocks with varying crystallographic misorientation angles, which may cause scattering in the texture due to differences in crystallographic orientations [61]. This feature is observed by the increase in misorientation angles and the increase in the fraction of HAGBs.

The geometrically necessary dislocation density $\rho_{GND}$ can be estimated using the KAM values:

(3) $$\rho_{GND}≅\frac{2\theta_{KAM}}{bd}$$

where *b* is the Burgers vector magnitude (0.255 nm for fcc iron), *d* is the step size (1 μm), and θ is the *KAM* misorientation angle [62]. *KAM* values of 1.41° and 1.35° were obtained for the undeformed samples, and thus, their $\rho_{GND}$ was determined to be 1.10 × 10^16^ m^−2^ and 1.06 × 10^16^ m^−2^, respectively. The KAM values and the determined *GND* densities suggest that the pre-existence of dislocation densities within the cell walls was likely generated by the rapid cooling and solidification process [54]. The pre-existence of these dislocation densities introduces obstacles to dislocations’ motion, and thus, more applied stress will be required for further deformation. The result is an increase in yield strength and improved ductility [35].

The dislocation densities measured for the L-PBF 316L stainless steel samples were higher than those of the conventionally manufactured materials in the literature. Odnobokova et al. [63] reported a dislocation density of 2 × 10^12^ m^−2^ for austenitic 316L stainless steel hot forged at 1373 K with a subsequent yield strength of 235 MPa and UTS of 585 MPa. Feaugas and Haddou [64] obtained dislocation densities below 10^10^ m^−2^ for AISI 316L stainless steel that was cold drawn and recrystallized to a grain size that ranged from 13 μm to 168 μm. Therefore, the high-density dislocations were observed to have also contributed to higher mechanical properties.

The continuous trapping of dislocations by the grain boundaries and the subdivision of grains to achieve strain compatibility eventually transform the LAGBs into HAGBs [59]. This is consistent with the grain boundary maps obtained for undeformed samples (Figure 8) and deformed samples (Figure 19) showing increased HAGBs after deformation. The formation of HAGBs enables the material to undergo further plastic deformation before a final fracture occurs.

The tensile test results showed an increase in the strength and ductility of L-PBF samples. This is different from conventionally manufactured stainless steel, where an increase in strength causes a reduction in ductility [42]. The absence of a tradeoff between strength and ductility in the L-PBF samples can be attributed to twinning. As the samples continue to deform, multiple slip systems are activated. Dislocation slips and deformation twinning are known as the two competing and complimentary deformation mechanisms for austenitic stainless steels [36]. Dislocation slip is usually the dominating mechanism in austenitic stainless steels. Depending on the crystallographic orientation and the stacking fault energy, the deformation twinning can be favored over the dislocation slip, more so with the increase in dislocation trapping and storage [65].

Twinning-induced plasticity (TWIP) is usually observed in steels that have more than 15% manganese and low stacking fault energy between 20 and 40 mJ/m^2^ at room temperature [44]. The chemical composition analysis of the as-built samples for both thicknesses confirmed that the Mn amounts of the samples were within the standard amounts of austenitic stainless steels, namely, <2%, where 1.94 wt.% and 1.66 wt.% were measured for the 1.5 mm and 4.0 mm, respectively. The average nitrogen amount, however, was slightly higher than the standard amounts and could be attributed to the nitrogen atmosphere used to fabricate the samples. The average amounts were 1.89 wt.% and 1.53 wt.% for the 1.5 mm and 4.0 mm thick samples, respectively. Nitrogen can significantly unlock dislocations, as it reduces the stacking fault energy, while at the same time stabilizing the austenitic phase in steels. Pham et al. [51] explained that low stacking fault energy promotes twinning formation in high nitrogen steels. Woo et al. [50] measured an average stacking fault energy of 32.8 mJ/m^2^ for additively manufactured 316L stainless steel, whereas Shamsujjoha et al. [63] estimated the stacking fault energy of additively manufactured 316L stainless steel to be 32 mJ/m^2^. According to Frommeyer et al. [66], stacking fault energy lower than 16 mJ/m^2^ results in a martensitic transformation, whereas twinning occurs when the stacking fault energy is higher than 25 mJ/m^2^. Thus, based on literature values of additively manufactured components, it can be inferred the slightly increased nitrogen wt.% could have reduced the stacking fault energy, thus encouraging the formation of twins, and deformation twinning became favored over dislocation slips.

Low stacking fault energy results in the dissociation of lattice dislocations into Shockley partial dislocations and stacking faults are formed [10]. These partial dislocations cannot cross-slip since the Burgers vector only belongs to the {111} plane [53]. The reduction in stacking fault energy results in a shift from a planar dislocation slip to stacking faults and finally to the formation of twins [67]. Liu et al. [16] explained that the pre-existing dislocation networks promote the formation of a high density of nano-twins during plastic deformation. As a result, the twin boundaries formed hinder dislocation motions by reducing the free mean path of dislocations on the slip planes that intersect with the twinning planes. Additionally, they cause a slip discontinuity due to the mirror symmetry that then delays the deformation of the materials. This enhances their strength and ductility, hence the absence of a tradeoff in the strength and ductility of the L-PBF 316L stainless steel samples tested.

Therefore, based on the in-depth microstructural analysis of the L-PBF 316L stainless steel samples, it can be concluded that the cellular structures, the fine grain sizes, the presence of LAGBs, and the formation of twins and HAGBs due to plastic deformation significantly contributed to the enhanced mechanical properties observed. The thorough microstructural characterization of the two thicknesses paves the way for further studies to be conducted on a wider range of thicknesses to understand the effect of size on various material properties. The understanding of these properties will further open the path to the production of L-PBF samples with enhanced properties and optimized geometries for various structural applications.

## 5. Conclusions

This study thoroughly examined the microstructural properties of L-PBF 316L stainless steel samples. Based on the findings, the following conclusions were inferred:

- In their undeformed states, the two thicknesses showed similar microstructural features indicating the use of well-optimized fabrication parameters. The optimal parameters can, therefore, be identified to be quite reliable if intricate and thin features need to be fabricated in one build platform.
- The YS, UTS, and elongation to failure of both thicknesses were observed to be significantly higher than conventionally manufactured 316L stainless steel samples. The 1.5 mm thick sample demonstrated a YS of 538.42 MPa, UTS of 606.47 MPa, and elongation to failure of 69.88%, whereas the 4.0 mm thick sample had a YS of 551.21 MPa, UTS of 619.58 MPa, and elongation to failure of 73.66%.
- The observed differences in the mechanical properties could be attributed to the difference in heat dissipation rates between the two thicknesses. Due to the different cooling rates, slight variations in microstructures were formed at the sub-micron level that ultimately resulted in different mechanical properties.
- The high tensile properties observed for both thicknesses were attributed to the cellular and columnar dendritic structures, with sizes ranging from 0.2 to 1 μm. These fine structures acted as strengthening mechanisms by hindering the motion of dislocations during plastic deformation. Additionally, the high percentage of LAGBs hindered the dislocation motion, thereby enhancing the strength of the materials. Also, high-density dislocations of 1.18 × 10^16^ m^−2^ and 1.41 × 10^16^ m^−2^ for the 1.5 mm and 4.0 mm thick samples, respectively, were observed. The accumulation of dislocations at the grain boundaries led to lattice distortion and strain incompatibilities. Therefore, more stress was required to overcome the resistance to deformation, and thus, the enhanced strain-hardening behavior of the material.
- Another crucial observation made was that upon deformation of the samples, the texture of the samples changed with thickness. Therefore, further studies need to be conducted to understand how the texture transitions as the thickness changes.

## Figures and Tables

**Table 1:** Chemical composition of 316L stainless steel powder (wt.%).

Element	Fe	Cr	Ni	Mo	Mn	Si	N	O	P	C	S
L-PBF Powder	Bal.	18.2	10.2	2.57	1.1	0.8	0.07	0.02	0.009	0.013	0.001
ASTM A240	Bal.	16–18	10–14	2–3	2.0	0.75	0.1	-	0.045	0.03	0.03

**Table 2:** L-PBF process parameters.

Laser Power	Scan Strategy	Layer Thickness	Hatch Distance	Hatch Exposure Time
180 W	Stripes	40 μm	85 μm	86 μs

**Table 3:** Chemical composition of the L-PBF 316L stainless steel samples.

Element	Fe	Cr	Ni	Mo	Mn	Si	N	O
1.5 mm	Bal.	16.43	10.42	0.82	1.94	0.28	1.89	2.59
4.0 mm	Bal.	16.16	10.55	0.88	1.66	0.18	1.53	2.47

**Table 4:** Summary of L-PBF 316L stainless steel tensile properties compared with ASTM 240/240-20a [28] 316L stainless steel.

Sample	YS (MPa)	UTS (MPa)	Ef (%)	Young’s Modulus (GPa)	Hardness (HRB)
1.5 mm	538.42 ± 4.26	606.47 ± 2.07	69.88 ± 2.27	156.52	87 ± 0.5
4.0 mm	551.21 ± 2.43	619.58 ± 3.23	73.66 ± 3.02	156.63	93 ± 1.5
ASTM A240	170	485	40	193	95

## Data Availability

The data that supports the findings of this study are available from the author upon request.
